# American Dental Association

**Published:** 1893-05

**Authors:** 


### Reports of Society Meetings.

### AMERICAN DENTAL ASSOCIATION.

(Concluded from page 281.)

*Fourth Day.—Morning Session.*

THE meeting was called to order by the president, Dr. Walker, after which the secretary read the names of the members of the different sections.

On motion of Dr. Crouse, the report of the committee that visited Washington in regard to the census law was called for.

Dr. John B. Rich, of Washington, responded as follows:

"Some time ago there was an attempt, on the part of the Census Bureau, to enforce part of their original bill in relation to dentists. Finding that by the terms of that bill they were not able to punish those who refused to answer the questions of the enumerators, they introduced into Congress a bill to punish them severely for failing to answer,—individuals as well as corporations,—and it was so carefully drawn that it passed through the House of Representatives without any one noticing its purport. When dentists refused to answer these questions, the census officials told them distinctly that in a little while they would be compelled to do so. It entered into every man's business,—the value of each tooth, how much he used each year, and every minute detail. It was on account of the peculiar inquisitorial character of this bill that the dentists of Maryland were aroused and started this movement.

"The attention of the society was called in May last to the bill known as the Wilcox Census Bill, which gave the Secretary of the Interior and his subordinates the authority to include dentists in the schedule of manufacturers, and to compel them to give detailed statements of their entire business. Upon a careful inspection of this bill, it became apparent that it was a most outrageous attempt on the part of the Census Bureau to establish a legal inquisition over whomsoever they pleased, and, as the Census Bureau had already issued these questions to many dentists who refused to answer, it was very evident that the menace which was made to those who had thus refused meant something, and had not been an idle ceremony.

"The society resolved to resist this action of the Census Bureau. They therefore appointed a committee to attend to the matter, and the following gentlemen were named: Drs. Winder, Nelson, Waters, Volck, Mills, Genise, and Twilley. Every means possible was to be used to prevent the passage of this bill, and to have it so modified that it could not be applied to dentists. They were to confer with other State societies, and get committees to co-operate in producing the desired result. The committee entered upon its duties at once. A large number of copies of the bill were printed, with the objectionable part underscored, which, with a written circular, was forwarded to dentists in every State, urging them to use their influence to prevent the passage of the bill. Dr. Winder prepared an eloquent argument against the bill. A copy of this argument was sent to all the members of Congress, and was largely instrumental in educating them regarding the proper status of the dental profession.

"Dr. Winder then personally laid the matter before all the State societies he could reach, and communicated with the others. As a result of these exertions the State societies of New York, New Jersey, Pennsylvania, Maryland, North Carolina, and the District of Columbia appointed committees to attend to the matter. They met in Washington and agreed to organize under the name of the 'Associated Committees of the Dental Societies,' and work together for the objects for which they were appointed. They elected Dr. Rich chairman of the body. By this time the bill had passed the House and was in the hands of the Committee on Census.

"The following amendment was then ordered: '*Provided*, That nothing in this act shall be construed as authority to collect statistics from professional men, such as lawyers, physicians, and dentists.'

"They directed their chairman, Dr. Rich, to ask for a hearing before the Senate Committee on the Census, in relation to having this amendment added to the bill, and if the application were granted to present the case on behalf of the dentists, to notify the members of the committee of the time of the hearing, so that as many as possible might be present.

"Dr. Rich applied to the chairman of the Committee on the Census for a hearing. This was accorded to him on June 27. In the mean time the chairman had written and sent to the different committees a circular, accompanied by copies of the amendment that the committee had adopted, and calling upon them to use all their influence with Congress, and the Senate in particular, to have the amendment introduced in the bill. Dr. Rich endeavored in every way to defeat the bill, in case the committee would not be in favor of the amendment. In this endeavor he was more successful than he had hoped to be. A Republican senator, to whom he presented the matter, became very much interested, and promised that if the bill should be reported to the Senate without the amendment, he would move to have it referred to the Judicial Committee, on the ground of its being unconstitutional. He also procured the assurance of a large number of Democratic senators in favor of the bill. At the hearing Dr. Rich presented the case for the dentists, and urged the insertion of the amendment presented. Argument on both sides of the question took place, and it looked as though the committee favored us. Superintendent Porter favored our views, and he finally said he did not wish to do anything that would place the dentists in a false position. Dr. Rich answered that if the superintendent would sign a statement, the dentists would be satisfied with such assurance, instead of having the amendment introduced into the bill. He agreed to this, and said that if Dr. Rich would come to his office the matter would be arranged; but Dr. Rich wished the matter arranged at once, in the presence of the Committee on Census. He then stated to the committee his understanding of the bill. Superintendent Porter signed the agreement on behalf of the committee. He then went to the Senate chamber, after the matter had been satisfactorily settled. The committee therefore beg to report that they were as successful in their resistance of this proceeding on the part of the superintendent of the Board of Census as could be hoped. The Associated Committees respectfully suggest that copies of the act be distributed to the dentists all over the country, so they may know what has been done for them, and as they have given this matter some attention, the committees suggest that the same be printed in a circular of four pages. They have had an estimate made of the cost of such a circular, with the result that ten thousand copies could be obtained on good paper for forty-eight dollars."

Dr. Shepard moved that the report be received and referred to the Publication Committee.

Adopted.

*Dr. Shepard.*—On behalf of the Executive Committee, I would offer the following preamble and resolutions:

WHEREAS, Under a classification of dentists as manufacturers by the Census Bureau many members of the profession have made reports according to such classification; and

WHEREAS, Such classification was the subject of protest by the profession at large, resulting in the following proposed amendment to House Bill No. 7696, known as the Wilcox bill,—"*Provided*, That nothing in this act shall be construed as authority to collect statistics from professional men, such as lawyers, physicians, and dentists;" and

WHEREAS, As a result of a conference between the representatives of the dental profession and the Committee on the Census of the United States (Senate) and the Hon. Mr. Porter, superintendent of the census, an agreement was made that if the committee representing the interests of the profession would withdraw the amendment to the Wilcox bill he would carry out the spirit of the provisions of the proposed amendment, with the following agreement: "The superintendent agrees with the representatives of the committees to carry out the spirit of the agreement."

*Resolved*, That the American Dental Association recommends its members, and the members of all societies affiliated with it who have made reports under the foregoing classification, to forward to the Hon. Robert P. Porter a request to return to the writer his statement made to the census authorities, so that the same shall cease to be a public record.

*Resolved*, That this Association wishes to express its appreciation of the promptness and public spirit of the Maryland State Dental Society, the New Jersey State Dental Society, the Odontological Society of Pennsylvania, and other societies, in attending to this important matter for the benefit of the whole profession.

*Resolved*, That our thanks are tendered to Dr. Rich for his valuable assistance, and that an order be drawn on the treasurer to reimburse him for expenses incurred, as per memorandum hereto attached.

*Resolved*, That full power be given to the Executive Committee to act for the Association in this matter in the interim of this meeting and the meeting of 1893.

For the benefit of the members I will state that the amount expended by Dr. Rich is about fifty-three dollars.

*Dr. Fillebrown.*—Why is it not competent that this Association demand that the authorities return those statements without any further action?

*Dr. Shepard.*—As a matter of opinion, it seems to be a general demand, and not a personal one. A personal demand is a request which must be answered personally; a general demand can be thrown into the waste basket; so it is better to have each one write personally.

*Dr. Crouse.*—The Executive Committee has had this matter before them twice, and we think it wise to put it in a form that will do the most good.

*Dr. Taft.*—The superintendent of the census ought to be very thankful to return to us our schedules. If this matter had been delayed two days the bill would have been killed, because it intended to bring pressure on all the chambers of commerce and manufacturing industries in the country. Our chamber of commerce had appointed a committee to go to Washington, and if these gentlemen had not made this promise the fate of the bill would have been settled.

Motion carried to adopt resolution.

*Dr. Rich.*—Does the Association want the custody of this agreement made with Superintendent Porter? Mr. Porter considers that the statistics which he already has he is entitled to publish. I have had an interview with him since the signing of this agreement, and he still thinks he has the right to publish these statistics. In a long talk I had with him he said, "Write to me, and I will tell you what I will do." I said I would not do so until the matter had been brought before the American Dental Association. I said, "You must bear in mind every member of Congress has some relation with dentists, and if you get those twenty thousand men buzzing about you, you will find yourself in a hornet's nest." He made the argument that he had been put to considerable expense in collecting these statistics, and he had a number of letters from dentists who said they would just as soon give these statistics as not. I said, "If there are men in my profession who are willing to be classed as manufacturers, I think they should be classed with blacksmiths. I want you to class us with professional men, where we belong." While he does not want to publish these statements, he wants to make as good a showing as possible for the moneys which he has expended for the government.

*Dr. Barrett.*—It seems to me that it would be proper for the secretary of the Association to be the custodian of the paper, and yet I can readily understand the necessity of Dr. Rich having it, in case he needs it. It is a document which should be preserved in the archives of this Association.

*Dr. Shepard.*—I move that this document be accepted by the Association, with thanks.

Carried.

On motion of Dr. Barrett, the secretary was instructed to place this where Dr. Rich can secure a certified copy when needed.

*Dr. Rich.*—What does the Association want me to do about the matter?

On motion of Dr. Crawford, it was decided that one of the duties of Dr. Rich shall be to visit the superintendent of the census bureau and request him to return to the members all statements which have been given in by them.

Motion carried.

Dr. Shepard moved that Dr. Rich be appointed a committee of one from this Association to act as its representative at Washington. A scheme for sending out this letter has come to my mind. They could be sent out in the same envelope with the notice of the World's Columbian Congress, and not cost any more.

Motion carried.

*Dr. Crouse.*—In reading the minutes last evening, there was a record of a resolution passed here in regard to excluding exhibits from any place near the meetings of this society. I would like an explanation from the originators of this movement.

*The President.*—I offered that resolution personally, for this reason. It seemed to me that it was beneath the dignity of the president of this Association to come here at the appointed time and find but one member of the Association present. I think it was hardly the proper thing for me to go out into the hall and request the dentists to come in and help me open the meeting. It seemed they preferred to remain outside and examine mechanical appliances to be seen elsewhere. The change has been made in the Dental Society of the State of New York. The exhibits detracted from the attention of the meeting; it was almost impossible to get the members into the hall to attend to the business for which they were supposed to be in attendance; and since we have eliminated those exhibits we have had satisfactory meetings. I thought that as long as the plan worked very well there it would do so here. We are here to listen to papers and aid in advancing scientific and practical research, and not to look at exhibits of dental instruments and manufactures of all kinds.

*Dr. Crouse.*—If some one will move a reconsideration, I will state my reasons for speaking about this.

Dr. Smith moved a reconsideration, which motion was carried.

*Dr. Crouse.*—In making the arrangements for this meeting, the chairman of the Executive Committee instructed the local committee to state, in the contract for this hall with the dental-supply companies, that they should close their doors at the hour of our meetings and keep them closed. If we had not had them here, they would have gone to the hotels, and we would have had no meeting at all until ten or eleven o'clock. When this society gives us something that is worth hearing, and warranting us in paying the expenses of coming here, then the members will come. The supply men were perfectly willing to close the doors. If the exhibits were at the hotels, you could not get a meeting at all. I would like to have these men have their exhibits right in the room of the meeting, and then when the meeting opens have them curtain them off. If the members would rather look at dental instruments, it is because they do not care for the work of the society. Do not pull down, but build up! I had the agreement in writing, and they were willing to close their doors when the meeting opened. At Excelsior Springs and wherever we have had this condition of things it has been the same. If you want the profession to be here in numbers, you must give them something worth coming for. If the profession would get these matters systematized, the reports and meetings of the society would be so interesting that we would have to hire a much larger hall to hold the audience.

*Dr. Barrett.*—Why do we have these meetings,—for the benefit of the profession or for the supply people? Those supply houses do not want to come here. This year the S. S. White Company has not sent any goods to sell at all; they prefer not to, but, of course, if one company comes the rest must do so. If some resolution could be passed, the dental companies would not come with their goods. There are many persons who wish to buy goods. That is not the real purpose of these meetings. This is something higher and nobler, and those who are here for the purpose of examining dental instruments are of no benefit to us. They pay their dues, but they do not come unless the meeting is somewhere in their neighborhood. They attract others and keep up a continual talk in the outside rooms. It is not because I am inimical to the supply houses; it is for their benefit as well as for ours. We shall never get the papers that we ought to have as long as the Association is conducted on the present plan. That has been tried for the past twenty years, and this Association has not filled the place it should occupy. We have not the intelligence of the profession here as we should have. I do not mean to say we have not the intelligent men, but I mean we have not all those who should be here. The present president of the Society wrote to the supply houses and asked them if they would stay away from the meeting of the Dental Society of the State of New York. They said that was just what they wanted ; but that if one house came they would all have to come. On the programme it was stated that no dental-supply houses would be represented there. We had a better meeting, more members, the finances are in a better condition, and the whole thing is decidedly improved. The present condition of the American Dental Association, with one hundred members present out of twenty thousand, is not the highest point to which this Association should reach. We do not come here to make money. It is not to peddle out privileges for selling goods. From the experience of the Dental Society of the State of New York, it is time to try something new and adopt the plan that they adopted with such great success. Every man who has been there can see the difference. I am not speaking against the supply houses, but I am in favor of trying another experiment, and of requesting that they should keep away from us.

*Dr. Allport.*—There is a sentimental and a practical side to this question. Every one comes to this Association for the purpose of being educated or to educate other people. Many come here with supplies that we perhaps know all about, and while I am opposed to it, there is a class of exhibits that should be made which is educational in its nature. It makes no difference to me or to Dr. Crouse whether they are brought or not, because we live in a large city where we can see these things all the time; but it makes a great difference to many of the country dentists, who come for hundreds of miles to attend these meetings. It is entirely proper that these things should be exhibited here for the benefit of those gentlemen, not to buy a gross of teeth or half a dozen forceps, but for the new articles that are constantly being brought out. Let the rule be established that these places shall be closed at the time of opening our meeting. If they are not closed, it is because the president and the officers of the Association do not perform their duty, although it is an unpleasant one. If they do not live up to their contract, the supply men forfeit it. We want those things as much as we need papers. Go to a medical convention, and you will find exhibits of all kinds. They are a source of great education. I think that the chairman of this committee is exactly right, and while I agree with Dr. Barrett that we should have better papers, I do not see why the exhibits should be barred out.

*Dr. Watkins.*—When we came here to attend this meeting, we came for different purposes. As Dr. Allport said, when new articles are brought into the profession, it is just as necessary that we should learn what they are as it is to hear many of the papers which are read. I want to agree entirely with Dr. Crousé, and emphasize what he said in regard to the exhibits. I think in New Jersey we have as good meetings as in any State of the Union. The best meetings we have ever had have been where the exhibits were in the room where the meeting was held. Before the meeting was called to order the exhibits were covered, and there was nothing to be seen during the meeting. Consequently the people had to come to the meeting or leave the room. That rule can be carried out here, or anywhere else, if the exhibit is near the place where the meeting is held. If it is at a distance, it cannot be done; they must be closely connected.

*Dr. Darby.*—Dr. Barrett said that the American Dental Association did not draw together the intelligent and representative men of the profession. I would take exception to that remark. I think it does. I would ask Dr. Barrett to name half a dozen intelligent, representative men of the profession who are not present to-day.

*Dr. Barrett.*—I stated that this did not represent all of the intelligence that it should. I expressly stated that I did not desire to make any reflection on those who were here, but there is no member present who cannot think of a great many men who ought to be here. There are one hundred and fifty members present out of fifteen or twenty thousand, and that is not a fair representation of the intelligence. There should be five hundred or six hundred at the very least.

Reconsideration carried.

Motion made that the original resolution be laid on the table, which was also carried.

Dr. Taft, of the Committee on Necrology, reported resolutions on the death of Dr. John Allen. (Published in vol. xiii. page 844.)

Dr. Harlan offered a resolution of thanks to such railroads as had given reduced rates to the members of the Association, and also a vote of thanks to the chairman of the local committee, Dr. Cooley.

Resolution adopted.

*Dr. Crouse.*—I wish to speak of the Dental Protective Association, and urge the members to take hold of this work and carry it out in their respective States. There is now a test case in progress in regard to the bridge patents, and all testimony that is possible should be presented to the Protective Association. It must not be thought, however, that when that case is finished our work will be ended. Many patents will be fought. If the profession would join as a whole the Association, the labor would be materially lessened. If we could get ten thousand members in the next four years, it would be a great gain, not only in the money received, but in the union of all, and if I should then need any testimony, I could readily write to our members and get it.

The Auditing Committee reported that they examined the treasurer's accounts for the past year and found them correct.

Report adopted.

The election of officers then took place, Drs. Ottofy and Thompson having been appointed as tellers, with the following result: President, Dr. J. D. Patterson; First Vice-President, Dr. J. Y. Crawford; Second Vice-President, Dr. S. C. G. Watkins; Corresponding Secretary, Dr. Fred. A. Levy; Recording Secretary, Dr. Geo. H. Cushing; Treasurer, Dr. A. H. Fuller.

*Executive Committee.*—Dr. S. G. Perry, Dr. W. W. Walker, Dr. D. N. McQuillan.

The final reading of the minutes then took place, and Drs. Thomas and McQuillan were appointed to conduct the newly-elected president to the chair.

Dr. Patterson accepted the office with pleasure, and made a few remarks, in which he stated that he had the welfare of the Association very much at heart, and would do all in his power to bring it up to a high standard.

Dr. Patterson then announced that the Publication Committee would consist of Drs. A. W. Harlan and E. T. Darby.

Adjourned, to meet in Chicago in 1893.

